# Salivary Telomere Length and Lung Function in Adolescents Born Very Preterm: A Prospective Multicenter Study

**DOI:** 10.1371/journal.pone.0136123

**Published:** 2015-09-10

**Authors:** Alice Hadchouel, Laetitia Marchand-Martin, Marie-Laure Franco-Montoya, Laetitia Peaudecerf, Pierre-Yves Ancel, Christophe Delacourt, EPIPAGEADO study group

**Affiliations:** 1 AP-HP, Hôpital Universitaire Necker-Enfants Malades, Pneumologie et Allergologie Pédiatriques, Paris, 75015, France; 2 INSERM, U955, équipe 4, Créteil, 94000, France; 3 Université Paris Descartes-Sorbonne Paris Cité, Paris, 75006, France; 4 INSERM, UMR 1153, Paris, 75004, France; 5 AP-HP, Unité de Recherche Clinique Cochin-Necker, Paris, 75015, France; University of Giessen Lung Center, GERMANY

## Abstract

Preterm birth is associated with abnormal respiratory functions throughout life. The mechanisms underlying these long-term consequences are still unclear. Shortening of telomeres was associated with many conditions, such as chronic obstructive pulmonary disease. We aimed to search for an association between telomere length and lung function in adolescents born preterm. Lung function and telomere length were measured in 236 adolescents born preterm and 38 born full-term from the longitudinal EPIPAGE cohort. Associations between telomere length and spirometric indices were tested in univariate and multivariate models accounting for confounding factors in the study population. Airflows were significantly lower in adolescents born preterm than controls; forced expiratory volume in one second was 12% lower in the extremely preterm born group than controls (p<0.001). Lower birth weight, bronchopulmonary dysplasia and postnatal sepsis were significantly associated with lower airflow values. Gender was the only factor that was significantly associated with telomere length. Telomere length correlated with forced expiratory flow 25–75 in the extremely preterm adolescent group in univariate and multivariate analyses (p = 0.01 and p = 0.02, respectively). We evidenced an association between telomere length and abnormal airflow in a population of adolescents born extremely preterm. There was no evident association with perinatal events. This suggests other involved factors, such as a continuing airway oxidative stress leading to persistent inflammation and altered lung function, ultimately increasing susceptibility to chronic obstructive pulmonary disease.

## Introduction

The respiratory consequences of preterm birth are not limited to the neonatal period and preterm birth is associated with respiratory morbidity throughout life. Children and young adults born very or extremely preterm [1,2], even those who did not develop bronchopulmonary dysplasia (BPD), suffer persistently impaired lung function. Airflow limitation is the main finding: the forced expiratory volume in 1 second (FEV_1_) is lower in preterm-born survivors than term-born controls [1], and even lower among those with BPD [1]. Unfortunately, this poor lung function persists throughout life [3], with no evidence of catch-up among ex-preterm individuals [2]. This raises the possibility of an increased risk of chronic obstructive pulmonary disease (COPD) in this population. The mechanisms underlying these long-term consequences are still unclear. It is not known if the observed airflow limitation results from a smaller than normal airway caliber, due to disrupted pulmonary growth [4], or continuing airway inflammation. Exhaled nitric oxide levels in ex-preterm children or adolescents were found to be normal or low [4–6]. However, a recent study evidenced higher levels of 8-isoprostane in exhaled breath condensates (EBC) of ex-premature adolescents, regardless of BPD status, than of healthy controls born at term [7]. This suggests a persistent oxidative stress and the existence of an ongoing disease in the airways of prematurely born adolescents [7]. Oxidative stress may have various consequences including effects on the regulation of cellular aging [8], in part by inducing a shortening of telomeres [9]. Telomeres are considered to be robust biomarkers of cellular replicative senescence and their length have been found to be associated with various aging-related diseases including cancers [10], neurodegenerative disorders [10], coronary-heart diseases [11] and type 2 diabetes [12]. Several studies found that telomere length (TL) is also associated with COPD, suggesting an accelerated aging process in the development of the disease [13–15]. In this setting, it was shown that telomere dysfunction perpetuates lung inflammation [16]. TL is positively associated with the pre-bronchodilatator values of spirometric indices FEV_1_, FVC (forced vital capacity) and FEV_1_/FVC, independently of any pathological respiratory status [17]. This suggests that lung function decline partially reflects biological aging due to intrinsic processes, with an aggravating role of diseases such as COPD and asthma [17]. In keeping with these studies performed in adults, and because there is an increasing evidence that adult lung function is driven by early life events [3,18], we built the following hypothesis: one of the mechanisms of the long-term respiratory consequences of premature birth could be an ongoing and dynamic oxidative stress that would, among others, lead to a shortening of telomeres and an accelerated replicative senescence. Shortening of telomeres *per se* may then prompt and maintain an inflammatory response. Consequently, we measured TL in the prospective EPIPAGE (Etude EPIdémiologique sur les Petits Ages Gestationnels) cohort of adolescents born very preterm and looked for correlations between the findings and spirometric indices in this population.

## Methods

### Study design and population

The study was approved by the local ethics committee (comité de protection des personnes, CPP Île-de-France VI). Study population was part of the EPIPAGE cohort, used for a prospective observational population-based study including all births between 22 and 32 completed weeks of gestation, and two reference groups of children born between 33 and 34, and 39 and 40 weeks of gestation, respectively, in 1997 in nine French region [19,20]. The methods of the EPIPAGE study are detailed in the smethods section of the S1 File. The study reported herein was restricted to the children born very preterm and at full term in Paris, Normandie, Pays-de-la-Loire, and Midi-Pyrénées regions. The inclusion criteria were complete participation in EPIPAGE from birth, assessment at 5 or 8 years old available, social insurance, and parental written informed consent. Subjects were prospectively included from November 2011 to June 2013.

### Lung function tests (LFT)

LFT were performed according to the recommendations of the American Thoracic Society/European Respiratory Society task force [21]. FVC, FEV_1_, functional residual capacity by plethysmography (FRC), total lung capacity (TLC), residual volume (RV), forced expiratory flow 50 (FEF50) and forced expiratory flow 25–75 (FEF25-75) were measured.

### Telomere length measurement

Saliva was collected with an Oragene DNA Self-Collection Kit OG-500 (DNA Genotek, Kanata, Ontario, Canada). Genomic DNA was extracted according to the manufacturer’s protocol and quantified with a spectrophotometer. TL was assessed by a real-time quantitative polymerase chain reaction (PCR)-based assay as previously described [22]. Details of the methods are given in the methods section of the S1 File and in S1 Fig.

### Statistical analyses

Data are expressed as medians and interquartile ranges (IQR). GLI 2012 lung function regression equations from the Global Lungs Initiative/ERS Task Force (TF-2009-03) (www.lungfunction.org) were used to compute % of predicted and Z-score values of spirometric indices, except for the FEF50 values. Principal analyses were performed using Z-score values according to recent guidelines [23,24]. These measures were analyzed as quantitative variables and as qualitative variables with two different thresholds for the lower limits of normal: -1.64 [23,24] and -1.96 Z-score [25], corresponding respectively to the 5^th^ and 3^rd^ percentiles of the distribution. FEF50 values are expressed as % of those predicted according to Zapletal [26] and were analyzed as quantitative variables. TL was analyzed as a quantitative variable. The subjects born preterm were divided into two groups according to their gestational age (GA): one group born between 24 and 28 (extremely preterm) and one group born between 29 and 32 completed weeks of gestation (very preterm). The results of LFT and TL were analyzed according to GA. Then, crude associations between TL and maternal and perinatal characteristics were studied. A similar analysis was conducted to identify maternal and perinatal factors associated with LFT. Correlations between TL and LFT, according to GA, were then studied using Pearson’s correlation coefficient. Linear regression models were used to quantify relationships between each lung function parameter and TL before and after adjustment for potential confounders. Confounders were variables selected on the basis of the results of the univariate analysis (p < 0,05): birth weight, sex, BPD and postnatal sepsis; and additionally smoking during pregnancy because of its known effects on lung function in offspring [27–29]. In linear regression models, the beta-coefficient for each LFT was estimated for an increase of 0.1 in TL.

## Results

### Study population (Fig 1)

In the four regions included in the study, 985 subjects were eligible to participate, including 805 ex-very-preterm individuals and 180 term-born controls. Lung function tests, written consent and saliva samples were obtained for 290 subjects. The main perinatal data did not differ between included and not included subjects, except for gender for the term-born controls, with more girls among the included term-born controls, and for ethnic maternal origin for the preterm-born subjects (Table A in S1 File). After DNA and PCR quality control procedures, 16 samples were excluded. Therefore, 274 adolescents, 236 ex-preterm individuals and 38 term-born controls, with a median age of 14.9 years old [14.7:15.3] were analyzed. Among the preterm-born subjects, 16.1% were diagnosed with BPD. Perinatal features by GA group are detailed in Table 1.

**Fig 1:**
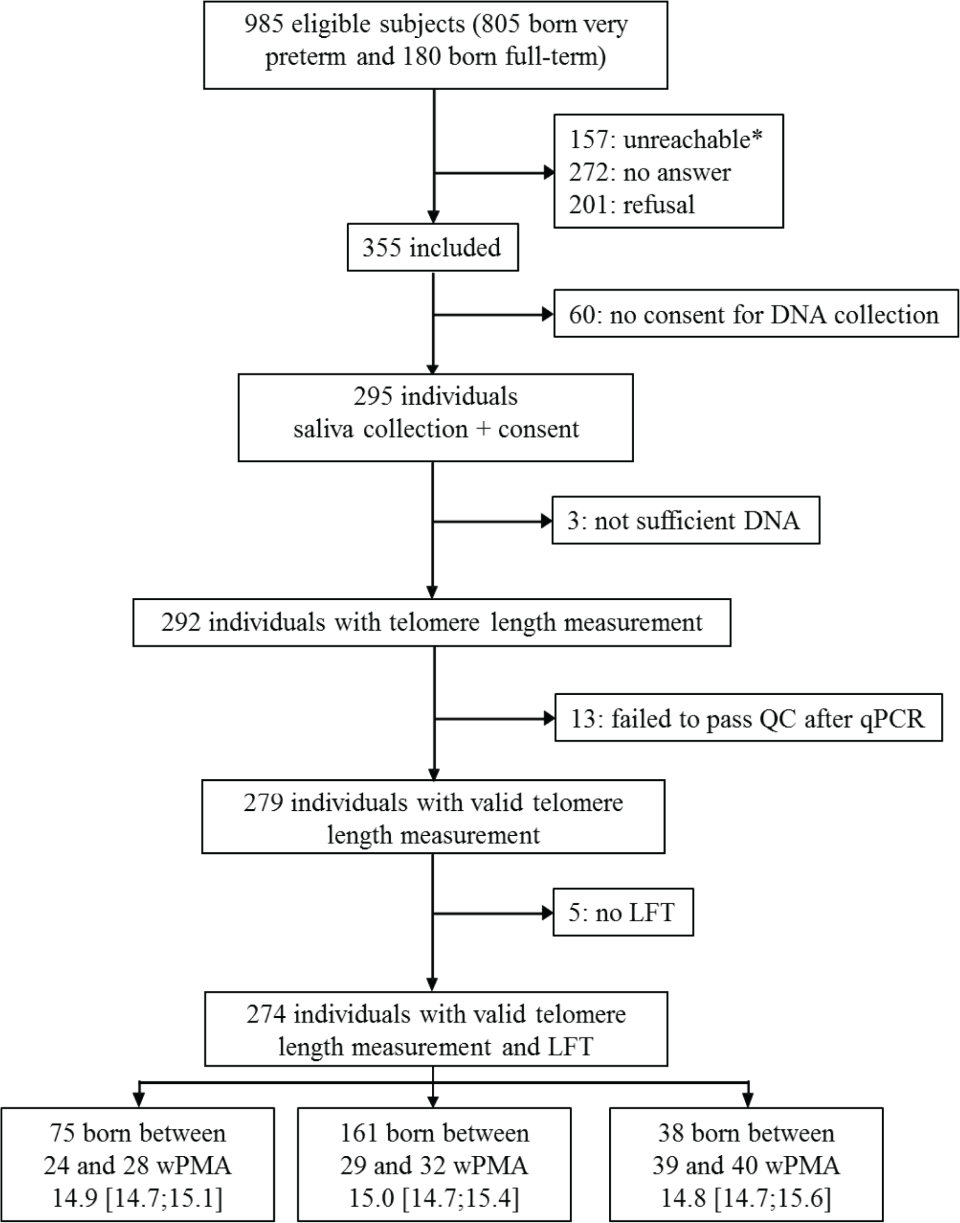
Flowchart of the study population. For each group, age at the time of the study is expressed as medians [IQR]. *probable move without leaving a forwarding address. LFT: lung function tests; QC: quality control; qPCR: quantitative polymerase chain reaction; wPMA: weeks of post-menstrual age.

**Table 1:** Epidemiological and clinical characteristics of the study population.

	24–28 w PMA		29–32 w PMA		24–32 w PMA		39–40 w PMA	
	n = 75		n = 161		n = 236		n = 38	
Maternal age at delivery								
<25 years old	7	(10)	18	(11)	25	(10.8)	5	(13)
25–34 years old	45	(62)	108	(68)	153	(65.9)	28	(74)
≥ 35 years old	20	(28)	34	(21)	54	(23.3)	5	(13)
NA	3		1		4		0	
Ethnic origin								
Caucasian	61	(81)	138	(85)	199	(84.3)	36	(95)
African	9	(12)	19	(12)	28	(11.9)	0	(0)
Asian	0	(0)	1	(1)	1	(0.4)	0	(0)
Mixed	5	(7)	3	(2)	8	(3.4)	2	(5)
Maternal smoking during pregnancy								
No	67	(89)	127	(79)	194	(82.2)	31	(82)
Yes	8	(11)	34	(21)	42	(17.8)	7	(18)
Sex								
Male	35	(47)	85	(53)	120	(50.8)	13	(34)
Female	40	(53)	76	(47)	116	(49.2)	25	(66)
Birth weight Z-score	-0.24 [-0.96;0.64]		-0.74 [-1.90;0.22]		-0.57 [-1.59;0.46]		0.41 [-0.33;0.88]	
BPD								
No	48	(64.0)	150	(93)	198	(84)		
Yes	27	(36.0)	11	(7)	38	(16)		

Continuous variables are expressed as medians [IQR] and categorical variables as numbers (%).

NA: not available data

### LFT and perinatal features among ex-preterm adolescents

Results of LFT in GA groups are presented in Table 2. Airflows (FEV_1_, FEF25-75 and FEF50) were significantly lower in preterm-born adolescents than term-born controls, with the lowest values in the extremely preterm-born group (Table 2). The proportions of children with FEV_1_, FEV_1_/FVC, or FEF25-75 Z-score values below -1.64 increased significantly with decreasing GA. Up to 16% of extremely preterm-born children but none of the term-born controls (p = 0.036) had an obstructive pattern defined as a FEV_1_/FVC Z-score below -1.64. Expressing LFT values as percentages of predicted values did not change the results (Table B in S1 File). FEV_1_ in the extremely preterm born group was 12% lower than in controls (Table B in S1 File). We tested for perinatal factors associated with the LFT measures FEV_1_, FEF25-75 and FEF50 in ex-preterm adolescents (Table C in S1 File). Lower birth weight, BPD and the occurrence of postnatal sepsis were significantly associated with lower airflow values (except for FEF50 and postnatal sepsis).

**Table 2:** LFT and telomere length according to group of gestational age.

		24–28 w PMA		29–32 w PMA		24–32 w PMA		39–40 w PMA	
		n = 75		n = 161		n = 236		n = 38	Global p*
LFT									
Z-score FEV_1_	75	-0.91 [-1.59;0.06]	161	-0.38 [-1.25;0.35]	236	-0.46 [-1.37;0.25]	38	0.10 [-0.86;0.39]	0.001
<-1.64 (%)	17	(23)	24	(15)	41	(17.4)	1	(3)	0.020†
<-1.96 (%)	12	(17)	16	(10)	28	(12.6)	1	(3)	0.067†
Z-score FVC	75	-0.42 [-1.26;0.33]	161	-0.32 [-1.08;0.43]	236	-0.33 [-1.14;0.43]	38	-0.11 [-0.78;0.36]	0.23
Z-score FEV_1_/FVC	75	-0.52 [-1.33;0.34]	161	-0.30 [-1.05;0.85]	236	-0.31 [-1.10;0.70]	38	0.04 [-0.70;1.12]	0.067
<-1.64 (%)	12	(16)	18	(11)	30	(13)	0	(0)	0.036†
<-1.96 (%)	8	(11)	12	(8)	20	(9)	0	(0)	0.11†
Z-score FEF25-75	75	-0.80 [-1.51;-0.09]	161	-0.33 [-1.08;0.46]	236	-0.49 [-1.33;0.26]	38	0.16 [-0.66;0.90]	< .001
Z-score<-1.64 (%)	15	(20)	28	(17)	43	(18)	0	(0)	0.014†
Z-score<-1.96 (%)	9	(13)	19	(12)	28	(13)	0	(0)	0.067†
FEF50 (% predicted)	75	89.5 [76.2;104.7]	161	98.5 [79.9;118.7]	236	95.5 [78.8;113.8]	38	105.9 [90.8;125.9]	0.001
Telomere length	75	0.99 [0.77;1.16]	161	0.95 [0.8;1.14]	236	0.97 [0.79;1.15]	38	0.9 [0.8;1.2]	0.68

Continuous variables are shown as medians [IQR] and categorical variables are shown as numbers (%).

*Comparison between the 24–28 wPMA, 29–32 wPMA and 39–40 wPMA groups with chi-square test or Kruskal-Wallis test, as appropriate.

†Compared to the normal control group (Z-score ≥ -1.64)

### Telomere length and perinatal features among ex-preterm adolescents

There was no difference of TL between the three groups of GA, or between the group of ex-preterm adolescents as a whole and term-born control adolescents (Table 2). We searched for individual and perinatal factors associated with TL (Table 3). Sex was the only factor that was significantly associated with TL, with longer telomeres in girls than in boys (p = 0.01).

**Table 3:** Telomere length and perinatal history among ex-preterm adolescents.

	n = 236	Telomere length	p*
		Median [IQR]	
**Maternal age at delivery**			
<25 years old	25	0.94 [0.77;1.15]	0.97
25–34 years old	153	0.95 [0.79;1.13]	
≥ 35 years old	54	0.99 [0.82;1.13]	
**Ethnic origin**			
Caucasian	199	0.95 [0.78;1.14]	0.25
African	28	0.99 [0.88;1.18]	
Asian	1	0.73 [0.73;0.73]	
Mixed	8	1.12 [0.89;1.33]	
**Maternal smoking during pregnancy**			
No	194	0.97 [0.78;1.13]	0.77
<10 cigarettes	30	0.95 [0.80;1.16]	
≥10 cigarettes	12	0.96 [0.84;1.31]	
**Maternal obesity at 3** ^**rd**^ **trimester (BMI>30)**			
Yes	21	1.07 [0.88;1.27]	0.22
No	166	0.94 [0.78;1.12]	
**Maternal wellbeing during pregnancy**			
Feeling very well or quite well	165	0.95 [0.80;1.15]	0.83
Feeling quite bad or very bad	34	1.02 [0.67;1.14]	
**Sex**			
Male	120	0.95 [0.74;1.08]	0.015
Female	116	1.00 [0.82;1.26]	
**Gestational age at birth**			
24–28 w PMA	75	0.99 [0.77;1.16]	0.55
29–32 w PMA	161	0.95 [0.80;1.14]	
**Birth weight (BW)**			
<1000gr	64	1.06 [0.81;1.34]	0.090
[1000gr-1500gr[	95	0.93 [0.73;1.12]	
≥1500gr	77	0.95 [0.82;1.10]	
**BW Z-score**			
<-1.65	57	1.01 [0.78;1.16]	0.73
[-1.65;-0.58[	61	0.97 [0.86;1.12]	
[-0.58;0.48[	59	0.95 [0.71;1.13]	
> 0.48	59	0.95 [0.80;1.15]	
**SGA**			
Yes	71	0.95 [0.78;1.15]	0.95
No	165	0.97 [0.80;1.15]	
**Cause and risk factors of premature birth**			
*Induced*	102	0.96 [0.74;1.12]	0.44
*Spontaneous*	129	0.98 [0.82;1.16]	
*HBP*	67	0.95 [0.78;1.12]	0.65
Yes			
No	169	0.98 [0.80;1.16]	
*IUGR*	47	1.08 [0.82;1.21]	0.26
Yes			
No	180	0.95 [0.78;1.13]	
*GDM*	11	0.88 [0.69;0.99]	0.24
Yes			
No	225	0.98 [0.79;1.15]	
*PRM*	103	0.98 [0.80;1.15]	0.37
Yes			
No	124	0.92 [0.77;1.14]	
**BPD**			
No	198	0.95 [0.79;1.15]	0.64
Yes	38	1.03 [0.74;1.16]	
**Postnatal sepsis**			
No	155	0.95 [0.80;1.12]	0.45
Yes	79	0.98 [0.77;1.25]	
**Necrotizing enterocolitis**			
No	229	0.97 [0.80;1.15]	0.94
Yes	6	0.86 [0.73;1.51]	

w PMA: weeks of post-menstrual age; SGA: small for gestational age defined by a birth weight below the 10^th^ percentile; HBP: high blood pressure; IUGR: intra-uterine growth retardation; GDM: gestational diabetes mellitus; PRM: premature rupture of membranes.

*Kruskal-Wallis test.

### Telomere length and lung function in ex-preterm adolescents

In univariate analyses, TL was associated with FEF25-75 and FEF50 in the extremely preterm adolescents, as shown by linear regression results (p = 0.01 and 0.03, respectively, Table 4). Airflows in this same group were analyzed as dichotomous variables, and FEF25-75 was associated with TL: median TL values were lower in individuals with FEF25-75 lower than -1.96 Z-score (p = 0.015, Table D in S1 File). TL was also lower, but not significantly, in the group of extremely preterm adolescents with FEV_1_ lower than -1.64 Z-score or lower than -1.96 Z score, (p = 0.053 and 0.057, respectively, Table D in S1 File).

**Table 4:** Telomere length and lung function parameters in ex-preterm adolescents.

	Telomere length								
	24–28 w PMA			29–32 w PMA			24–32 w PMA		
	N	Pearson's Rho	p	N	Pearson's Rho	p	N	Pearson's Rho	p
Z-score FEV_1_	75	0.19	0.10	161	0.02	0.82	236	0.06	0.33
Z-score FEV_1_/FVC	75	0.08	0.47	161	0.10	0.22	236	0.09	0.19
Z-score FEF25-75	75	0.29	0.011	161	0.04	0.58	236	0.11	0.09
FEF50% predicted	75	0.25	0.029	161	-0.004	0.96	236	0.06	0.32

After adjustment for potential confounding factors by multiple linear regression, TL remained correlated to FEF25-75 in the extremely preterm adolescent group (p = 0.02, Table 5).

**Table 5:** Telomere length and lung function parameters before and after adjustment for potential confounders.

	24–28 w PMA			29–32 w PMA		24–32 w PMA			
	N	Coeff* (95%CI)	p	N	Coeff* (95%CI)	p	N	Coeff* (95%CI)	p
T/S ratio	Z-score FEV1								
Crude	75	0.07 (-0.01;0.15)	0.11	161	0.01 (-0.06;0.07)	0.82	236	0.03 (-0.03;0.08)	0.33
Adjusted	74	0.08 (-0.01;0.17)	0.09	160	0.02 (-0.05;0.08)	0.62	234	0.04 (-0.01;0.09)	0.11
	z-score FEV1/FVC								
Crude	75	0.03 (-0.06;0.12)	0.47	161	0.04 (-0.03;0.12)	0.22	236	0.04 (-0.02;0.09)	0.19
Adjusted	74	0.04 (-0.06;0.14)	0.45	160	0.06 (-0.01;0.13)	0.090	234	0.06 (0.00;0.11)	0.039
	Z-score FEF25-75								
Crude	75	0.10 (0.02;0.17)	0.011	161	0.02 (-0.05;0.09)	0.58	236	0.04 (-0.01;0.09)	0.094
Adjusted	74	0.09 (0.02;0.17)	0.020	160	0.02 (-0.04;0.09)	0.47	234	0.06 (0.01;0.11)	0.030
	FEF50% predicted								
Crude	75	1.8 (0.2;3.3)	0.029	161	-0.0 (-1.5;1.4)	0.96	236	0.6 (-0.5;1.7)	0.32
Adjusted	74	1.5 (-0.1;3.1)	0.071	160	0.1 (-1.4;1.5)	0.92	234	0.8 (-0.3;1.9)	0.18

*Coefficient for an increase of 0.1 in telomere length

Adjustment for birth weight, BPD, sex, postnatal sepsis and maternal cigarette smoking during pregnancy

## Discussion

We report a significant association between distal airflows and TL in extremely preterm-born teenagers, which remained significant after adjustment for potential confounding factors. Our study also confirmed that preterm birth is associated with long-term impairment of lung function, in agreement with previous reports [1]. As far as we know, this is the first analysis of the potential association between lung function and TL in a prospective cohort of ex-preterm adolescents. TL was significantly correlated with FEF25-75. The correlation between FEV_1_ and TL was very close to being significant, although lower FEV_1_ values were also related to shorter telomeres. FEF25-75 is usually considered to be a more sensitive index than FEV_1_ of airway obstruction in children [30]. Although our population is sufficiently numerous to be one of the most powerful long-term prospective cohort of premature infants available, the number of children included in this study may have been too small to identify associations with FEV_1_. Like many other similar cohorts, the number of evaluable patients decreased over time. Families moving away, or becoming weary with the protocol, led to substantial loss to follow-up. Nevertheless, our results suggest that shortening of telomeres are independently involved in the origin of the lung function impairments observed in adolescents that were born extremely preterm.

Numerous studies have addressed cellular aging biomarkers in various diseases. We chose TL measurement by quantitative PCR because it is the most commonly used marker in this setting [10–15]. In our study, DNA was extracted from saliva whereas most studies use DNA extracted from blood leucocytes or buffy coat. We chose this sample type because of its non-invasiveness and its better acceptance than blood sampling by teenagers. Approximately 80% of the cells in saliva are lymphocytes, and a study that used both DNA from saliva and blood leucocytes from a cohort of children showed a correlation between TL from saliva and peripheral blood of 0.96 (p < 0.001) [31]. A correlation was also found between TL obtained from peripheral blood and lung tissue in a study on pulmonary fibrosis [32]. These results validate our non-invasive approach.

In our population, telomeres were significantly longer in girls than in boys, in agreement with previous reports [33,34]. We also sought an association between TL and ethnicity, previous data being contradictory on this issue (reviewed in [34]). No such association was found in our population. We found no association between gestational age at birth or perinatal events and TL, suggesting that preterm birth *per se* and its associated short-term complications are not risk factors for shortening of telomeres. Diverging results were published about the impact of pregnancy duration, birth weight and other perinatal factors on TL, which could be explained by differences in participant populations and methodology. Indeed, some studies investigated TL in neonates whereas others measured TL in children or adults born preterm or small for gestational age (SGA). As in our study, Kajantie et al. did not find any association between TL and GA at birth in 3 independent Finnish cohorts, including one large birth cohort of 1894 adults [35]. Laganovic et al. found longer telomeres in adults in the first quartile of birth weight and pregnancy duration than those in the fourth quartile in a population of 114 young men [36]. Conversely, Entringer et al. observed an independent positive association between birth weight adjusted for gestational age and TL in a population of 94 adults [37]. They also reported an independent relationship between prenatal stress and TL, with prenatal stress exposure predicting significantly shorter telomeres [37]. This association with maternal stress was published later by the same authors in newborn leukocytes, but at birth they did not observe an association between TL and birth weight [38]. Regarding other studies performed in neonates, Friedrich et al. found no difference in mean TL measured in cord blood leukocytes between preterm neonates and full-term newborns, but found longer telomeres in very low birth weight infants than low birth weight infants [39]. A more recent study measured TL in endothelial colony-forming cells that were isolated from the mononuclear cell fraction of cord blood from term and preterm neonates, and telomeres were not shorter in preterm cells than term cells [40]. Two other studies compared TL at birth owing to birth weight or fetal growth restriction. Akkad et al found no difference in cord-blood telomere length between babies born SGA and those born appropriate for gestational age in a population composed of moderate preterm (≥ 35 weeks) and full-term newborns [41]. Similar results were reported by Davy et al. who found no difference in cord-blood telomere length between fetal-growth-restricted and normal weight babies born full-term [42].

Our results reveal a complex interaction between shortening of telomeres and long-term functional respiratory outcome in preterm-born children. Preterm birth or its complications, such as BPD, do not themselves appear to cause telomere dysfunction, as suggested by the absence of association between them and TL; the significant association between TL and lung function is observed only in extremely preterm-born adolescents. This suggests that telomere dysfunction is active in extremely preterm born individuals, regardless of perinatal complications, and contribute to the degree of functional impairment. Although perinatal events surrounding preterm birth may lead to increased oxidative stress, potentially exacerbated by the immature anti-oxidant defense capacities of preterm newborns, our results also suggest that this mechanism does not appear to be sufficient to induce shortening of telomeres. Our findings therefore call into question one of the current hypotheses that abnormal lung functions only result from interactions between disruption of normal lung development and consequences of perinatal lung injuries [4]. Our work provides evidence that preterm birth *per se* may be an independent risk factor for a persistent biological stress throughout life that may lead to both continuing airway disease and inflammation, and also to a consecutive accelerated shortening of telomeres progressively later in life. The work performed by Filippone et al. corroborates our results [7]. In this study, oxidative stress was assessed in ex-preterm individuals by measuring the concentration of 8-isoprostane in exhaled breath condensates (EBC) [7]: 8-isoprostane is one of the most reliable biomarkers of oxidative stress *in vivo* [43,44]. Levels of 8-isoprostane exhaled by ex-preterm adolescents were higher than those exhaled by healthy controls born at term, suggesting a persistent oxidative stress in the airways of preterm born adolescents [7]. Like TL, the levels of 8-isoprostane were not related to perinatal events, consistent with preterm birth being an independent risk factor of long-term oxidative stress [7]. Oxidative stress was shown to induce shortening of telomeres in several experiments, *in vitro* (reviewed in [9]) and *in vivo* [45]. All of these results bring a new insight into the mechanisms of altered lung function in preterm-born individuals, even if it does not fully explain all of our results. In particular, 8-isoprostane levels did not correlate with the degree of preterm birth [7], whereas in our study the association between TL and lung function was significant only for extremely preterm-born adolescents. The small number of children participating in the study of isoprostane levels may have prevented identification of the influence, if any, of term at birth on long-term oxidative mechanisms. As lower GA is also associated with worse lung function, it is likely that long-term respiratory consequences of preterm birth are multifactorial and of complex origin: preterm birth itself, life-course events and possibly genetic factors in these individuals may all contribute. Finally, telomere dysfunction is able to induce an inflammatory response [16]. Compared to wild-type mice, inflammatory cytokines levels in lung tissue were increased in mice null for the telomerase reverse transcriptase (Tert) or telomerase RNA component (Terc) genes, despite the absence of external stimuli and in proportion to telomere dysfunction [16]. Shortening of telomeres in our population could thus contribute to a persistent inflammation in the airways. This hypothesis of a persistent oxidative stress and sustained inflammation is also supported by a recent study that compared metabolic profiles of EBC between adolescents with BPD and healthy controls using a metabolomic approach [46]. Two biomarkers, namely LPC and PAF, were increased in BPD subjects and are putative biomarkers of oxidative stress and inflammation respectively [46].

## Conclusion

We evidenced an association between telomere length and airflow abnormalities in a population of adolescents born extremely preterm. This accelerated shortening of telomeres do not seem to be associated with perinatal events, and suggest that other factors are involved, such as a persistent airway inflammation.

## Supporting Information

S1 Fig Distribution of *36B4* Ct values. This graph shows the distribution of *36B4* Ct values among all DNA samples: the distribution was nearly normal. The samples with extreme values were excluded from the analysis (2.5% on each side of the distribution).
(TIFF)

S1 File Supporting methods and tables A to D. Table A: Perinatal factors among included and non-included eligible subjects; Table B: LFT parameters expressed as %ages of predicted values according to group of gestational age; Table C: LFT parameters and perinatal history among ex-preterm adolescents; Table D: Telomere length and lung function parameters as dichotomous variables.
(DOCX)
