# Supplementary figures and images for: Salivary Telomere Length and Lung Function in Adolescents Born Very Preterm: A Prospective Multicenter Study
Source: PLoS One. 2015 Sep 10;10(9):e0136123. doi: 10.1371/journal.pone.0136123 (PMC4565668; doi:10.1371/journal.pone.0136123)

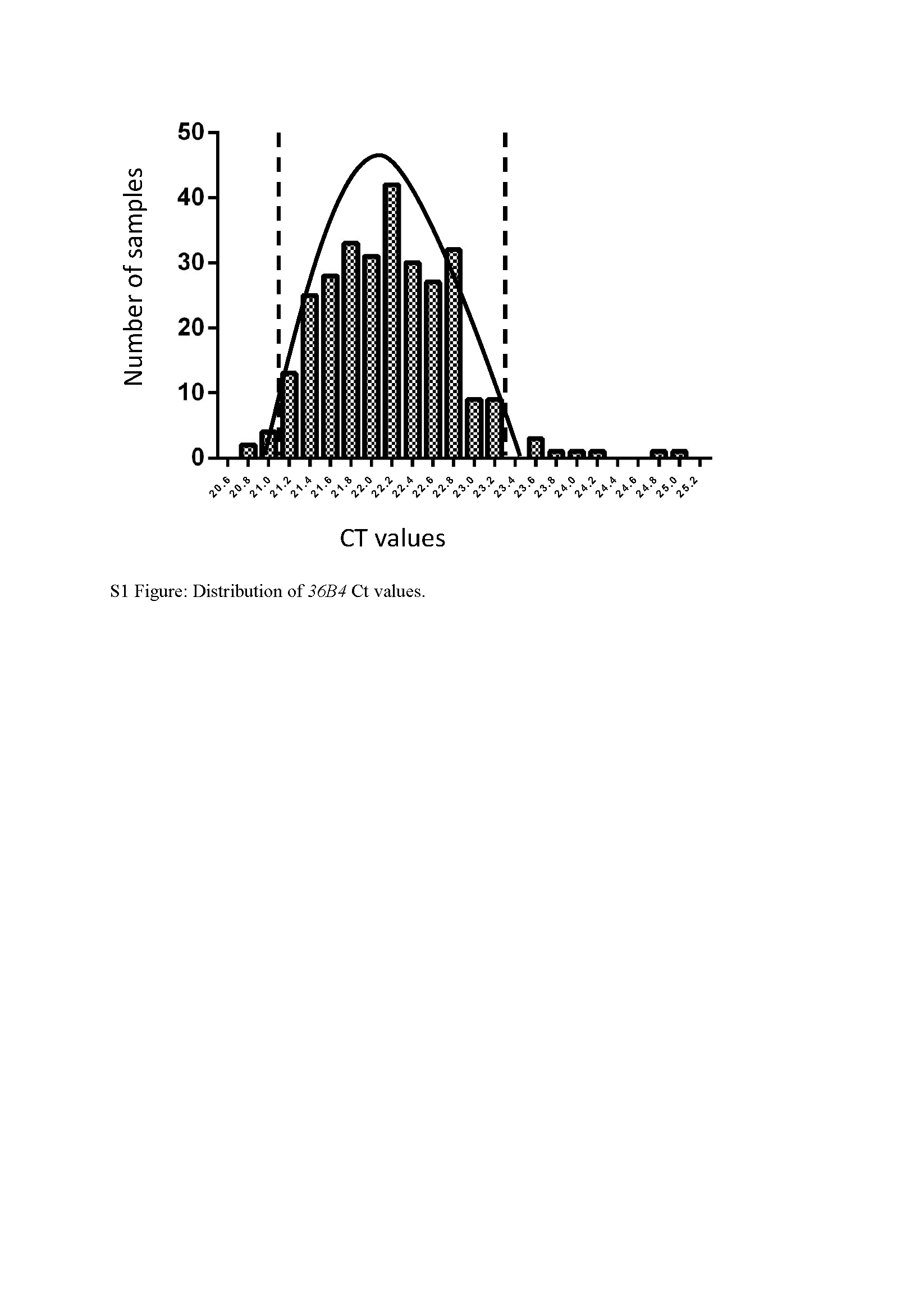

Supplement: S1 Fig — This graph shows the distribution of 36B4 Ct values among all DNA samples: the distribution was nearly normal. The samples with extreme values were excluded from the analysis (2.5% on each side of the distribution). (TIFF) [file pone.0136123.s001.tiff]
