# Supplementary material for: Salivary Telomere Length and Lung Function in Adolescents Born Very Preterm: A Prospective Multicenter Study
Source: PLoS One. 2015 Sep 10;10(9):e0136123. doi: 10.1371/journal.pone.0136123 (PMC4565668; doi:10.1371/journal.pone.0136123)
Supplement: S1 File — Table A: Perinatal factors among included and non-included eligible subjects; Table B: LFT parameters expressed as %ages of predicted values according to group of gestational age; Table C: LFT parameters and perinatal history among ex-preterm adolescents; Table D: Telomere length and lung function parameters as dichotomous variables. (DOCX) [file pone.0136123.s002.docx]

Salivary telomere length and lung function in adolecents born very preterm: a prospective multicenter study

Supporting methods and tables

**Methods**

**The EPIPAGE study**

EPIPAGE (Etude EPIdémiologique sur les Petits Ages Gestationnels) is a prospective observational population-based study that included all births (live births and stillbirths) and late terminations of pregnancy, occurring between 22 and 32 completed weeks of gestation, in 1997, to women in the maternity wards of nine French regions [1]. Two control groups were also included, one of -individuals born at term (one in every four births at 39 or 40 weeks of gestation during 1 week in 1997), and one of moderate preterm infants, born between 33 and 34 weeks of gestation (inclusion during 2 months in 1997). At recruitment in the maternity or neonatal unit, parents were told about the study and given written information, and oral consent was provided to the medical team in charge of the study. No written consent was obtained. The study was approved by the French Commission Nationale de l’Informatique et des Libertés (the French data protection agency). The objectives of the study were the following [2]:

- Description of obstetrical and neonatal practices for the care of very preterm newborns
- Analysis of the causes of very preterm birth
- Measures of neonatal mortality, and short- and long-term survival
- Evaluation of the children and their families during childhood
- Identification of the pre-, per- and postnatal factors involved in the health of the child and his/her development
- Study of the adverse effects of intensive care techniques and of the treatments used for very preterm newborns

Data for mothers, pregnancies, births, and neonatal outcomes were recorded on standardized questionnaires at each maternity and neonatal intensive care unit. Maternal data included ethnic origin, tobacco consumption during pregnancy, and causes of preterm birth. Neonatal data included gender, gestational age (determined from the last menstrual period and findings from early prenatal ultrasound scans, calculated in complete weeks), birth weight, postnatal sepsis defined as a postnatally acquired infection treated with antibiotics for at least 7 days, necrotizing enterocolitis and bronchopulmonary dysplasia (BDP). BPD was defined as the need for supplemental oxygen and/or ventilatory support at 36 weeks of post-menstrual age. Information about the health and the development of the children was subsequently collected by questionnaires sent to the families two months after discharge, and when the child was nine months old, and one, two, three and four years old. At 2 years of age, a questionnaire was also sent to the child’s physician. At 5 years of age, the children were invited for a check-up with a physician and a psychologist at local centers in every region. At 8 years of age, a new questionnaire was sent to the families.

Of the 2901 preterm liveborn children that were included from January 1, 1997, to December 31, 1997 [1], 2459 (85%) were alive at discharge. At age 5 years, of the 2251 survivors (95%) whose parents agreed to follow-up at birth, 1812 (80%) had a clinical evaluation [3]. At age 8 years, 1444 questionnaires (64%) were completed by families. In the full-term birth reference group, 666 children were included. Follow-up rates were 59% (n=396) at 5 years and 49% (n=327) at 8 years.

**Telomere length measurement**

Telomere length (TL) was measured by a real-time quantitative polymerase chain reaction assay [4]. The telomere repeat copy number to the single-copy gene *36B4* (encoding the acidic ribosomal phosphoprotein PO) copy number (T/S) ratio was determined with a 7900HT thermocycler (Applied Biosystems, Foster City, CA) in a 96-well format, using the comparative Ct method (T/S = 2^-ΔΔCt^). Each sample was run in triplicate, using the SYBR green method (Invitrogen, Cergy-Pontoise, France) and 30 ng of DNA. The sequences and final concentrations of the primers for the telomere and *36B4* were as follows: TelF, 5’-CGGTTTGTTTGGGTTTGGGTTTGGGTTTGGGTTTGGGTT-3’ 300 nM; TelR, 5’-GGCT TGCCTTACCCTTACCCTTACCCTTACCCTTACCCT-3’, 300 nM; 36B4F, 5’-CAGCA AGTGGGAAGGTGTAATCC-3’, 300 nM; and 36B4R, 5’-CCCATTCTATCATCAACGGGTACAA-3’, 300 nM. A quality control procedure was applied prior to statistical tests by analyzing the distribution among samples of raw Ct values obtained for the *36B4* gene; the values for this gene should be similar in all samples. The distribution of *36B4* Ct values was close to a normal distribution (S1 Fig.) and the samples with extreme values were excluded from the analysis (2.5% on each side of the distribution).

REFERENCES

1. Larroque B, Bréart G, Kaminski M, Dehan M, André M, Burguet A, et al. Survival of very preterm infants: Epipage, a population based cohort study. Arch Dis Child Fetal Neonatal Ed. 2004;89: F139–144.

2. Larroque B. [EPIPAGE: epidemiologic study of very premature infants. Protocol of the survey]. Arch Pédiatrie Organe Off Sociéte Fr Pédiatrie. 2000;7 Suppl 2: 339s–342s.

3. Larroque B, Ancel PY, Marret S, Marchand L, Andre M, Arnaud C, et al. Neurodevelopmental disabilities and special care of 5-year-old children born before 33 weeks of gestation (the EPIPAGE study): a longitudinal cohort study. Lancet. 2008;371: 813–20.

4. Cawthon RM. Telomere measurement by quantitative PCR. Nucleic Acids Res. 2002;30: e47.

**Supporting tables:**

**Table A: Perinatal factors among included and non-included eligible subjects**

|  | 24-32 w PMA | | | | 39-40 w PMA | | | |  |  |
| --- | --- | --- | --- | --- | --- | --- | --- | --- | --- | --- |
|  | Non included (1) | | Included (2) | | Non included (3) | | Included (4) | | p-value | p-value |
|  | n=569 | | n=236 | | n=142 | | n=38 | | (1)/(2) | (3)/(4) |
| **Maternal age at delivery** |  |  |  |  |  |  |  |  |  |  |
| <25 years old | 88 | (15.7) | 25 | (10.8) | 18 | (13) | 5 | (13) | 0.11 | 0.70 |
| 25-34 years old | 369 | (65.6) | 153 | (65.9) | 97 | (68) | 28 | (74) |  |  |
| >= 35 years old | 105 | (18.7) | 54 | (23.3) | 27 | (19) | 5 | (13) |  |  |
| NA | 7 |  | 4 |  |  |  |  |  |  |  |
| **Ethnic maternal origin** |  |  |  |  |  |  |  |  |  |  |
| Caucasian | 492 | (89.9) | 202 | (85.6) | 133 | (94) | 38 | (100) | 0.003 | 0.28 |
| African | 41 | (7.5) | 33 | (14.0) | 6 | (4) | 0 |  |  |  |
| Other | 14 | (2.6) | 1 | (0.4) | 3 | (2) | 0 |  |  |  |
| NA | 22 |  | 0 |  |  |  |  |  |  |  |
| **Maternal smoking during pregnancy** |  |  |  |  |  |  |  |  |  |  |
| No | 401 | (76) | 194 | (82.2) | 108 | (78) | 31 | (82) | 0.11 | 0.77 |
| <10 cigarettes | 81 | (15.3) | 30 | (12.7) | 21 | (15) | 4 | (10) |  |  |
| >=10 cigarettes | 46 | (8.7) | 12 | (5.1) | 10 | (7) | 3 | (8) |  |  |
| NA | 41 |  | 0 |  | 3 |  | 0 |  |  |  |
| **Sex** |  |  |  |  |  |  |  |  |  |  |
| Male | 280 | (49.2) | 120 | (50.8) | 84 | (59) | 13 | (34) | 0.67 | 0.006 |
| Female | 289 | (50.8) | 116 | (49.2) | 58 | (41) | 25 | (66) |  |  |
| **Gestational age at birth** |  |  |  |  |  |  |  |  |  |  |
| 24-28 w PMA | 162 | (28.5) | 75 | (31.8) |  |  |  |  | 0.35 |  |
| 29-32 w PMA | 407 | (71.5) | 161 | (68.2) |  |  | |  |  |  |
| **Birth weight (BW)** |  |  |  |  |  |  |  |  |  |  |
| <1000gr | 142 | (25.0) | 64 | (27.1) |  |  |  |  | 0.72 |  |
| [1000gr-1500gr[ | 226 | (39.7) | 95 | (40.3) |  |  |  |  |  |  |
| >=1500gr | 201 | (35.3) | 77 | (32.6) |  |  |  |  |  |  |
| **BW Z-score** |  |  |  |  |  |  |  |  |  |  |
| <-1.65 | 155 | (27.2) | 57 | (24.2) |  |  |  |  | 0.76 |  |
| [-1.65;-0.58[ | 132 | (23.2) | 61 | (25.8) |  |  |  |  |  |  |
| [-0.58;0.48[ | 137 | (24.1) | 59 | (25.0) |  |  |  |  |  |  |
| > 0.48 | 145 | (25.5) | 59 | (25.0) |  |  |  |  |  |  |
| **Cause of premature birth** |  |  |  |  |  |  |  |  |  |  |
| HBP/IUGR | 159 | (27.9) | 73 | (30.9) |  |  |  |  | 0.73 |  |
| Threatened preterm labor /PRM | 319 | (56.1) | 122 | (51.7) |  |  |  |  |  |  |
| Hemorrhage | 44 | (7.7) | 20 | (8.5) |  |  |  |  |  |  |
| Other | 47 | (8.3) | 21 | (8.9) |  |  |  |  |  |  |
| **BPD** |  |  |  |  |  |  |  |  |  |  |
| No | 473 | (85.1) | 198 | (83.9) |  |  |  |  | 0.67 |  |
| Yes | 83 | (14.9) | 38 | (16.1) |  |  |  |  |  |  |
| NA | 13 |  | 0 |  |  |  |  |  |  |  |
| **Postnatal sepsis** |  |  |  |  |  |  |  |  |  |  |
| No | 388 | (69.3) | 155 | (66.2) |  |  |  |  | 0.40 |  |
| Yes | 172 | (30.7) | 79 | (33.8) |  |  |  |  |  |  |
| NA | 9 |  | 2 |  |  |  |  |  |  |  |
| **Necrotizing enterocolitis** |  |  |  |  |  |  |  |  |  |  |
| No | 470 | (83.3) | 203 | (86.0) |  |  |  |  | 0.34 |  |
| Yes | 94 | (16.7) | 33 | (14.0) |  |  |  |  |  |  |
| NA | 5 |  | 0 |  |  |  |  |  |  |  |
| w PMA: weeks of post-menstrual age; HBP: high blood pressure; IUGR: intra-uterine growth retardation; PRM: premature rupture of membranes | | | | | | | | | | |

**Table B: LFT parameters expressed as %ages of predicted values according to group of gestational age**

|  | 24-28 w PMA | | 29-32 w PMA | | 24-32 w PMA | | 39-40 w PMA | | Global p* |
| --- | --- | --- | --- | --- | --- | --- | --- | --- | --- |
|  | n | Median [IQR] | n | Median [IQR] | n | Median [IQR] | n | Median [IQR] |  |
|  |  |  |  |  |  |  |  |  |  |
| FEV_1_ % predicted | 75 | 89.1 [81.2;100.7] | 161 | 95.4 [85.0;104.1] | 236 | 94.6 [83.9;103.1] | 38 | 101.2 [90.0;104.5] | <0.001 |
| < 80% (%) | 17 | (23) | 24 | (15) | 41 | (17) | 1 | (3) | 0.020 |
|  |  |  |  |  |  |  |  |  |  |
| FEV_1_/FVC | 75 | 0.86 [0.80;0.91] | 161 | 0.87 [0.82;0.93] | 236 | 0.86 [0.81;0.92] | 38 | 0.90 [0.85;0.94] | 0.068 |
| < 0.8 (%) | 18 | (24) | 32 | (20) | 50 | (21) | 4 | (10) | 0.23 |
|  |  |  |  |  |  |  |  |  |  |
| FEF25-75 % predicted | 75 | 83.0 [68.4;97.9] | 161 | 92.7 [77.3;111.0] | 236 | 89.7 [72.5;106.1] | 38 | 103.5 [86.1;121.1] | <0.001 |
| < 80% (%) | 34 | (45) | 50 | (31) | 84 | (36) | 7 | (18) | 0.011 |
|  |  |  |  |  |  |  |  |  |  |
| FEF50 < 80% (%) | 23 | (31) | 41 | (25) | 64 | (27) | 2 | (5) | 0.010 |

Continuous variables are shown as median [IQR] and categorial variables are shown as numbers (%).

*Comparison between the 3 groups 24-28 wPMA, 29-32 wPMA and 39-40 wPMA with chi-square test or Kruskal-Wallis test, as appropriate.

**Table C: LFT parameters and perinatal history among ex-preterm adolescents*.**

|  |  | Z-score FEV_1_ | | Z-score FEF25-75 | | FEF50 % predicted | |
| --- | --- | --- | --- | --- | --- | --- | --- |
|  | n=236 | median [IQR] | p† | median [IQR] | p† | median [IQR] | p† |
| Maternal age at delivery |  |  |  |  |  |  |  |
| <25 years old | 25 | -1.04 [-1.51;-0.09] | 0.16 | -1.01 [-1.79;-0.52] | 0.09 | 86.6 [69.7;104.9] | 0.25 |
| 25-34 years old | 153 | -0.43 [-1.28;0.27] |  | -0.43 [-1.19;0.20] |  | 95.3 [80.3;111.2] |  |
| >= 35 years old | 54 | -0.55 [-1.33;0.25] |  | -0.31 [-1.35;0.53] |  | 99.3 [79.2;116.6] |  |
| Maternal smoking  during pregnancy |  |  |  |  |  |  |  |
| No | 194 | -0.53 [-1.43;0.18] | 0.25 | -0.51 [-1.48;0.25] | 0.19 | 93.1 [77.5;111.2] | 0.21 |
| <10 cigarettes | 30 | -0.07 [-1.27;0.63] |  | -0.30 [-0.81;0.70] |  | 100.2 [86.3;125.1] |  |
| >=10 cigarettes | 12 | -0.67 [-1.19;0.66] |  | -0.31 [-0.98;-0.02] |  | 98.6 [79.6;116.8] |  |
| Gestational age at birth |  |  |  |  |  |  |  |
| 24-28 w PMA | 120 | -0.61 [-1.29;0.38] | 0.92 | -0.54 [-1.33;0.26] | 0.92 | 92.8 [78.9;113.5] | 0.91 |
| 29-32 w PMA | 116 | -0.44 [-1.43;0.18] |  | -0.45 [-1.31;0.26] |  | 97.1 [78.8;114.6] |  |
| Birth weight (BW) |  |  |  |  |  |  |  |
| <1000gr | 64 | -1.00 [-1.66;0.00] | 0.004 | -0.79 [-1.51;-0.02] | 0.04 | 89.4 [69.9;106.1] | 0.06 |
| [1000gr-1500gr[ | 95 | -0.43 [-1.41;0.35] |  | -0.55 [-1.35;0.25] |  | 93.4 [78.8;118.3] |  |
| >=1500gr | 77 | -0.20 [-1.03;0.42] |  | -0.27 [-0.88;0.46] |  | 100.4 [84.3;114.6] |  |
| BW Z-score |  |  |  |  |  |  |  |
| <-1.65 | 57 | -0.70 [-1.49;0.10] | 0.36 | -0.54 [-1.27;0.20] | 0.09 | 93.9 [78.8;113.7] | 0.07 |
| [-1.65;-0.58[ | 61 | -0.40 [-1.04;0.57] |  | -0.32 [-1.24;0.51] |  | 102.2 [82.5;117.9] |  |
| [-0.58;0.48[ | 59 | -0.46 [-1.44;0.18] |  | -0.81 [-1.73;-0.10] |  | 88.2 [73.7;103.8] |  |
| > 0.48 | 59 | -0.34 [-1.24;0.18] |  | -0.27 [-1.17;0.52] |  | 100.1 [78.9;119.4] |  |
| Cause of premature birth |  |  |  |  |  |  |  |
| HBP/IUGR | 73 | -0.53 [-1.33;0.18] | 0.91 | -0.45 [-1.17;0.42] | 0.74 | 97.5 [80.3;114.0] | 0.66 |
| Threatened  preterm labor/RPM | 122 | -0.46 [-1.41;0.18] |  | -0.40 [-1.35;0.15] |  | 96.1 [78.7;111.2] |  |
| Hemorrhage | 20 | -0.42 [-1.44;0.24] |  | -0.77 [-1.72;0.43] |  | 86.4 [69.7;111.6] |  |
| Other | 21 | -0.59 [-1.37;0.94] |  | -0.77 [-1.61;0.33] |  | 96.6 [74.7;133.8] |  |
| BPD |  |  |  |  |  |  |  |
| No | 198 | -0.35 [-1.25;0.31] | <.0001 | -0.36 [-1.05;0.43] | <.0001 | 98.5 [82.5;116.9] | <.0001 |
| Yes | 38 | -1.23 [-2.14;-0.73] |  | -1.39 [-1.96;-0.44] |  | 76.9 [63.5;93.0] |  |
| Postnatal sepsis |  |  |  |  |  |  |  |
| No | 155 | -0.29 [-1.27;0.27] | 0.01 | -0.39 [-1.08;0.42] | 0.05 | 97.7 [79.9;117.9] | 0.10 |
| Yes | 79 | -0.82 [-1.76;0.14] |  | -0.76 [-1.58;0.07] |  | 89.5 [74.0;112.6] |  |
| Necrotizing enterocolitis |  |  |  |  |  |  |  |
| No | 229 | -0.46 [-1.37;0.26] | 0.37 | -0.50 [-1.35;0.26] | 0.56 | 95.3 [78.8;113.7] | 0.84 |
| Yes | 6 | -1.10 [-2.10;0.14] |  | -0.73 [-1.00;-0.35] |  | 95.4 [74.0;116.6] |  |

*Ethnic origin and sex were not analyzed, because they were already taken into account by lung function regression equations.

†Kruskal-Wallis test.

**Table D: Telomere length and lung function parameters as dichotomous variables**

|  | **Telomere length (T/S ratio)** | | | | | | | | |
| --- | --- | --- | --- | --- | --- | --- | --- | --- | --- |
|  | **24-28 w PMA** | | | **29-32 w PMA** | | | **24-32 w PMA** | | |
|  | N | median [IQR] | p* | N | median [IQR] | p* | N | median [IQR] | p* |
|  |  |  |  |  |  |  |  |  |  |
| Z-score FEV_1_ |  |  |  |  |  |  |  |  |  |
| < -1.64 | 17 | 0.83 [0.68;1.04] | 0.053† | 24 | 0.95 [0.80;1.11] | 0.83† | 41 | 0.94 [0.75;1.08] | 0.32† |
| < -1.96 | 12 | 0.81 [0.66;1.01] | 0.057† | 16 | 0.95 [0.78;1.09] | 0.99† | 28 | 0.92 [0.74;1.07] | 0.25† |
| > -1.64 | 58 | 1.03 [0.82;1.25] |  | 137 | 0.95 [0.80;1.15] |  | 195 | 0.98 [0.80;1.16] |  |
|  |  |  |  |  |  |  |  |  |  |
| Z-score FEV_1_/FVC |  |  |  |  |  |  |  |  |  |
| < -1.64 | 12 | 0.86 [0.77;1.08] | 0.48† | 18 | 0.93 [0.82;1.08] | 0.61† | 30 | 0.92 [0.79;1.08] | 0.41† |
| < -1.96 | 8 | 0.93 [0.77;1.08] | 0.57† | 12 | 0.95 [0.85;1.07] | 0.88 | 20 | 0.95 [0.80;1.07] | 0.64† |
| > -1.64 | 63 | 1.01 [0.77;1.23] |  | 143 | 0.97 [0.79;1.15] |  | 206 | 0.98 [0.79;1.15] |  |
|  |  |  |  |  |  |  |  |  |  |
| Z-score FEF25-75 |  |  |  |  |  |  |  |  |  |
| < -1.64 | 15 | 0.83 [0.74;1.39] | 0.51† | 28 | 0.94 [0.80;1.08] | 0.64† | 43 | 0.94 [0.75;1.10] | 0.47† |
| < -1.96 | 9 | 0.75 [0.68;0.83] | 0.015† | 19 | 0.94 [0.82;1.08] | 0.63† | 28 | 0.90 [0.74;1.04] | 0.076† |
| > -1.64 | 60 | 1.00 [0.80;1.16] |  | 133 | 0.97 [0.80;1.15] |  | 193 | 0.98 [0.80;1.16] |  |
|  |  |  |  |  |  |  |  |  |  |
| FEF50 % predicted |  |  |  |  |  |  |  |  |  |
| <80% | 23 | 0.91 [0.75;1.25] | 0.61† | 41 | 0.97 [0.80;1.09] | 0.85† | 64 | 0.96 [0.79;1.10] | 0.67† |
| >80% | 52 | 1.02 [0.78;1.16] |  | 120 | 0.95 [0.79;1.16] |  | 172 | 0.97 [0.79;1.16] |  |
|  |  |  |  |  |  |  |  |  |  |
| *Kruskal-Wallis test | | | | | | | | | |
| †Compared to normal group (Z-score ≥ - 1.64 or > 80%) | | | | | | | | | |
